# Sepsis and the Risks of Long-Term Renal Adverse Outcomes in Patients With Chronic Kidney Disease

**DOI:** 10.3389/fmed.2022.809292

**Published:** 2022-02-24

**Authors:** Shuo-Ming Ou, Kuo-Hua Lee, Ming-Tsun Tsai, Wei-Cheng Tseng, Yuan-Chia Chu, Der-Cherng Tarng

**Affiliations:** ^1^Division of Nephrology, Department of Medicine, Taipei Veterans General Hospital, Taipei, Taiwan; ^2^School of Medicine, National Yang-Ming University, Taipei, Taiwan; ^3^School of Medicine, National Yang Ming Chiao Tung University, Taipei, Taiwan; ^4^Institute of Clinical Medicine, National Yang-Ming University, Taipei, Taiwan; ^5^Institute of Clinical Medicine, National Yang Ming Chiao Tung University, Taipei, Taiwan; ^6^Center for Intelligent Drug Systems and Smart Bio-Devices (IDS2B), National Yang Ming Chiao Tung University, Hsinchu, Taiwan; ^7^Information Management Office, Taipei Veterans General Hospital, Taipei City, Taiwan; ^8^Big Data Center, Taipei Veterans General Hospital, Taipei City, Taiwan; ^9^Department of Information Management, National Taipei University of Nursing and Health Sciences, Taipei, Taiwan; ^10^Department and Institute of Physiology, National Yang-Ming University, Taipei, Taiwan; ^11^Department and Institute of Physiology, National Yang Ming Chiao Tung University, Taipei, Taiwan

**Keywords:** sepsis, chronic kidney disease, AKI (acute kidney injury), renal function decline, end-stage renal disease

## Abstract

**Background:**

Sepsis is known to cause renal function fluctuations during hospitalization, but whether these patients discharged from sepsis were still at greater risks of long-term renal adverse outcomes remains unknown.

**Methods:**

From 2011 to 2018, we included 1,12,628 patients with chronic kidney disease (CKD) aged ≥ 20 years. The patients with CKD were further divided into 11,661 sepsis group and 1,00,967 non-sepsis group. The following outcome of interest was included: all-cause mortality, readmission for acute kidney injury, estimated glomerular filtration rate decline ≥50% or doubling of serum creatinine, and end-stage renal disease.

**Results:**

After propensity score matching, the sepsis group was at higher risks of all-cause mortality [hazard ratio (HR) 1.39, 95% CI, 1.31–1.47], readmission for acute kidney injury (HR 1.67, 95% CI 1.58–1.76), eGFR decline ≥ 50% or doubling of serum creatinine (HR 3.34, 95% CI 2.78–4.01), and end-stage renal disease (HR 1.43, 95% CI 1.34–1.53) than non-sepsis group.

**Conclusions:**

Our study found that patients with CKD discharged from hospitalization for sepsis have higher risks of subsequent renal adverse events.

## Introduction

Chronic kidney disease (CKD) is a global health burden with a prevalence of ~10–16% worldwide and a high economic cost (1–3). Because patients with CKD show a decline in renal function with time, the identification of modifiable risk factors for renal function decline, leading to early intervention and slow down of CKD progression and its associated complications (4, 5). The relatively immunocompromised status of patients with CKD could potentially predispose them to sepsis, which contributes to a higher risk of death and substantial morbidity (6–8). Patients with CKD who had a lower estimated glomerular filtration rate (eGFR) were also found to be at greater risks of infection than those who had a higher eGFR (9, 10).

Sepsis affects renal microcirculation due to hemodynamic instability, which causes acute tubular necrosis and renal cellular damage (11–13). Sepsis-inducing inflammatory cytokines have also been shown to be associated with the severity and worsening of renal function impairment (14, 15). Interestingly, plasma extracted from patients with septic still induced renal cell injury and renal tubular and podocyte apoptosis without the presence of vasculature or circulating inflammatory cells (16). Although there is increasing evidence that sepsis can increase the risk of acute kidney injury (AKI) (17–19), the relationship between sepsis and long-term renal adverse outcomes, especially in the fragile population with CKD, remains unclear.

To address this knowledge gap, we explored the association of sepsis and future risks of long-term all-cause mortality and renal adverse outcomes, including readmission for AKI, renal function decline, or development of end-stage renal disease (ESRD) by performing a large-scale CKD cohort study. In our study, competing risk analysis was also performed to account for mortality as a competing risk for renal adverse outcomes.

## Methods

### Study Design and Setting

In this study, data were retrieved from the electronic medical database of the Big Data Center at Taipei Veterans General Hospital. The datasets are de-identified for research purposes and contain basic demographic information, disease diagnoses, drug prescriptions, surgery records, and laboratory results from inpatient, outpatient, and emergency data (20). We established a CKD cohort by using diagnostic codes [International Classification of Diseases (ICD) code 581–583, 585–589, N00–N08, N18–N19, and N25–N27] from January 1, 2011, to December 31, 2018. We further categorized our patients with CKD into two groups as follows: (1) those who had a history of discharge from sepsis (ICD code 038, 995.91, A40, and A41), severe sepsis (ICD code 995.92 and R65.20), or septic shock (ICD code 785.52 and R65.21) as the sepsis group and (2) those without a history of hospitalization for sepsis as the non-sepsis group. In our study, we excluded patients aged < 20 years, those who received hemodialysis, peritoneal dialysis, or kidney transplant before they were eligible for inclusion, and those who did not have at least two measurements of serum creatinine values to assess the eGFR decline. Finally, 112,628 patients with CKD (11,661 in the sepsis group and 100,967 in the non-sepsis group) were included in our study. The study was approved by the institutional review board of Taipei Veterans General Hospital (2017-09-002BC) and informed consent was waived due to the de-identified data being analyzed.

### Clinical Covariates

The patient information obtained from the electronic medical database consisted of demographic characteristics, comorbidity histories, and medication prescriptions. The demographic characteristics were age, gender, smoking status, and alcohol consumption. Laboratory data such as hemoglobin, total cholesterol, glycated hemoglobin, eGFR, and the spot urine protein–creatinine ratio were also collected. The eGFR was estimated using the Chronic Kidney Disease Epidemiology Collaboration (CKD-EPI) equation (21, 22). Comorbidity histories consisted of hypertension, diabetes mellitus, coronary artery disease, congestive heart failure, peptic ulcer disease, chronic obstructive pulmonary disease, malignancy, and Charlson Comorbidity Index (CCI) score (23, 24). The medication prescriptions collected were for calcium channel blockers, beta-blockers, alpha-blockers, angiotensin-converting enzyme inhibitors/angiotensin II receptor blockers, antiplatelets, warfarins, statins, steroids, non-steroidal anti-inflammatory drugs, oral hypoglycemic agents, and insulins.

### Outcome Definition

The primary outcomes were all-cause mortality, readmission for AKI, eGFR decline ≥ 50% or doubling of serum creatinine, and ESRD (defined as eGFR < 15 ml/min/1.73 m^2^, initiation of long-term hemodialysis/peritoneal dialysis, or kidney transplantation). The readmission for AKI was defined based on the acute kidney injury network (AKIN) classification, which defines 3 stages of AKI: AKIN stage 1 classified as a ≥0.3 mg/dl absolute or 1.5- to 2.0-fold increase in serum creatinine from baseline; AKIN stage 2 as a 2- to 3-fold increase in serum creatinine, and AKIN stage 3 as a baseline serum creatinine > 4.0 mg/dl with an acute increase of ≥0.5 mg/dl or a >3-fold increase in serum creatinine or the initiation of renal replacement therapy (25, 26). The percent decline in eGFR is calculated as follows: (last eGFR at the follow-up—baseline eGFR)/(baseline eGFR) × 100% (27, 28). Patients with CKD were followed up until death or the end of the study period, whichever occurred first.

### Statistical Analysis

Data from continuous variables are presented as median (interquartile range [IQR]) and categorical data are presented as percentages (numbers). For missing values, we performed multiple imputations with five repetitions for handling (29). In addition, we calculated propensity scores for the likelihood of sepsis by including clinical covariates in a multivariate logistic regression model (Supplementary Table 1) (30, 31). For propensity score matching, we matched each sepsis group to one non-sepsis group on the basis of propensity scores using nearest-neighbor matching without replacement. Cox proportional hazards models were used to evaluate risks of all-cause mortality and other outcomes of interest in the sepsis group compared to the non-sepsis group. All statistical analyses were performed using SAS version 9.4 (SAS Institute, Cary, NC, United States) and R software (version 3.5.2 for Windows). A two-tailed *P* < 0.05 was considered statistically significant.

## Results

### The Incidence of Different Infection Sources Among Patients With CKD

**Figure 1:**
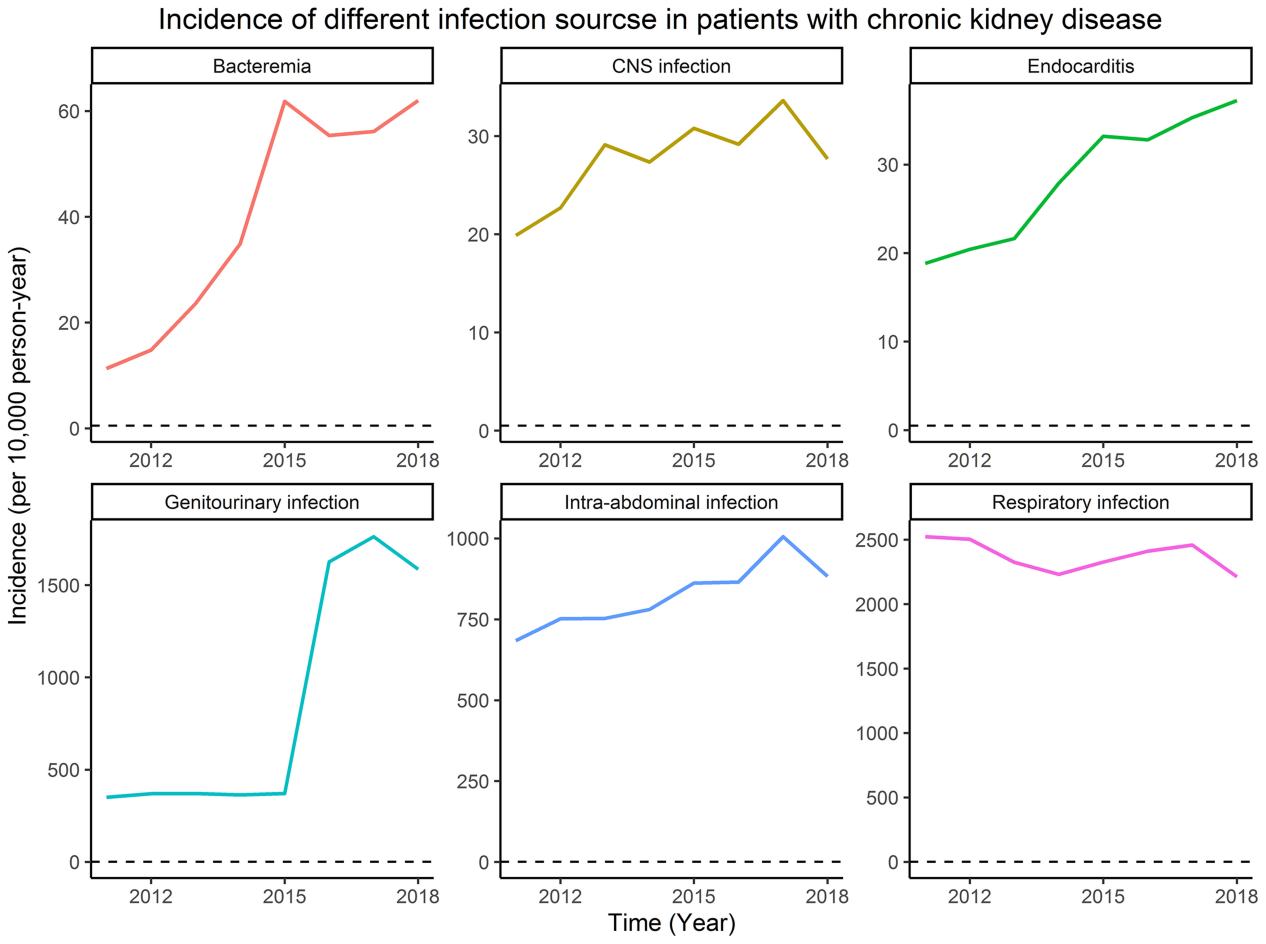
The incidence of different infection sources among patients with chronic kidney disease (CKD).

From January 1, 2011, to December 31, 2018, the different infection sources, including bacteremia, central nervous system infection, endocarditis, genitourinary infection, intra-abdominal infection, and respiratory infection among patients with CKD are shown in Figure 1. As the years evolve, the incidence of bacteremia, endocarditis, genitourinary infection, and intra-abdominal infection increased gradually. Of note, the incidences of genitourinary and respiratory infection were the highest two infection sources among patients with CKD.

### Study Population

A total of 112,628 CKD patients with a median age of 65.5 years (interquartile range: 55.0–77.9 years) were included in our study. Patients with CKD were then divided into sepsis and non-sepsis groups, and the detailed characteristics of the two cohorts are shown in Table 1. In the overall patient group, we identified 11,661 sepsis cases and 100,967 non-sepsis cases. The sepsis group was older and was more likely to be male, smoke, consume alcohol, had a history of diabetes mellitus, coronary artery disease, and congestive heart failure had a higher CCI score and had higher prescription rates of antihypertensive drugs, oral hypoglycemic agents (OHAs), and insulin. After propensity score matching, 9,336 sepsis groups and 9,336 non-sepsis groups were included in the analyses, and the baseline characteristics were well-balanced between these two groups (Supplementary Figure 1). The distributional balance of the propensity score before and after propensity score matching is shown in Supplementary Figure 2.

**Table 1:** Baseline characteristics of the study population before and after propensity score matching.

	**Before propensity score matching**				**After propensity score matching**			
	**All patients**	**Non-Sepsis group**	**Sepsis group**	**SMD**	**All patients**	**Non-Sepsis group**	**Sepsis group**	**SMD**
Clinical variables^*^	**(*****n*** **=** **112,628)**	**(*****n*** **=** **100,967)**	**(*****n*** **=** **11,661)**		**(*****n*** **=18,672)**	**(*****n*** **=** **9,336)**	**(*****n*** **=** **9,336)**	
Age, years	65.5 [55.0, 77.9]	64.5 [54.3, 76.5]	76.7 [63.3, 85.5]	0.590	75.0 [62.2, 83.9]	75.1 [62.8, 83.0]	74.9 [61.6, 84.7]	0.009
Male sex, *n* (%)	62,871 (55.8)	55,944 (55.4)	6,927 (59.4)	0.081	10,821 (58.0)	5,449 (58.4)	5,372 (57.5)	0.017
Smokers, *n* (%)	24,794 (22.0)	20,505 (20.3)	4,289 (36.8)	0.371	6,252 (33.5)	3,131 (33.5)	3,121 (33.4)	0.002
Alcohol consumption, *n* (%)	18,223 (16.2)	14,932 (14.8)	3,291 (28.2)	0.331	4,815 (25.8)	2,443 (26.2)	2,372 (25.4)	0.017
Hgb, g/dL	12.9 [11.4, 14.1]	13.1 [11.7, 14.3]	10.5 [9.3, 12.0]	1.099	11.0 [9.5, 12.5]	11.1 [9.4, 12.6]	10.8 [9.6, 12.3]	0.016
Total cholesterol, mg/dL	178.0 [152.0, 205.0]	179.0 [155.0, 206.0]	160.0 [134.0, 188.0]	0.430	164.0 [139.0, 191.0]	165.0 [140.0, 191.0]	163.5 [137.0, 192.0]	0.004
HbA_1c_, %	6.9 [6.1, 8.3]	6.9 [6.1, 8.2]	7.2 [6.1, 10.3]	0.092	7.0 [6.1, 9.0]	7.0 [6.2, 8.4]	7.1 [6.1, 10.0]	0.001
eGFR, mL/min/1.73 m^2^	76.7 [52.2, 93.7]	78.2 [54.9, 94.5]	58.2 [30.2, 83.1]	0.532	59.3 [33.3, 83.2]	57.9 [34.0, 81.9]	61.0 [32.3, 84.1]	0.038
UPCR, g/g	0.22 [0.09, 0.98]	0.21 [0.09, 0.90]	0.43 [0.13, 1.72]	0.025	0.36 [0.11, 1.56]	0.33 [0.11, 1.43]	0.40 [0.12, 1.66]	0.004
HTN, *n* (%)	45,485 (40.4)	37,945 (37.6)	7,540 (64.7)	0.563	11,004 (58.9)	5,476 (58.7)	5,528 (59.2)	0.011
Diabetes mellitus, *n* (%)	42,283 (37.5)	36,740 (36.4)	5,543 (47.5)	0.227	8,096 (43.4)	3,977 (42.6)	4,119 (44.1)	0.031
CAD, *n* (%)	19,264 (17.1)	15,688 (15.5)	3,576 (30.7)	0.365	4,854 (26.0)	2,429 (26.0)	2,425 (26.0)	0.001
CHF, *n* (%)	8,657 (7.7)	6,106 (6.0)	2,551 (21.9)	0.469	3,127 (16.7)	1,546 (16.6)	1,581 (16.9)	0.010
Peptic ulcer disease, *n* (%)	10,323 (9.2)	7,501 (7.4)	2,822 (24.2)	0.472	3,430 (18.4)	1,699 (18.2)	1,731 (18.5)	0.009
COPD, *n* (%)	7,359 (6.5)	5,092 (5.0)	2,267 (19.4)	0.450	2,620 (14.0)	1,315 (14.1)	1,305 (14.0)	0.003
Malignancy, *n* (%)	23,734 (21.1)	18,848 (18.7)	4,886 (41.9)	0.523	6,705 (35.9)	3,294 (35.3)	3,411 (36.5)	0.026
CCI score	3.0 [1.0, 4.0]	2.0 [1.0, 4.0]	4.0 [3.0, 6.0]	0.723	4.0 [2.0, 6.0]	4.0 [2.0, 6.0]	4.0 [2.0, 6.0]	0.012
CCB, *n* (%)	40,480 (35.9)	34,068 (33.7)	6,412 (55.0)	0.438	9,797 (52.5)	4,915 (52.6)	4,882 (52.3)	0.007
Beta blockers, *n* (%)	33,000 (29.3)	27,836 (27.6)	5,164 (44.3)	0.354	7,669 (41.1)	3,805 (40.8)	3,864 (41.4)	0.013
Alpha blockers, *n* (%)	19,229 (17.1)	15,557 (15.4)	3,672 (31.5)	0.387	5,425 (29.1)	2,732 (29.3)	2,693 (28.8)	0.009
ACEIs/ARBs, *n* (%)	42,359 (37.6)	36,649 (36.3)	5,710 (49.0)	0.258	8,788 (47.1)	4,388 (47.0)	4,400 (47.1)	0.003
Antiplatelets, *n* (%)	29,016 (25.8)	24,544 (24.3)	4,472 (38.4)	0.306	6,673 (35.7)	3,352 (35.9)	3,321 (35.6)	0.007
Warfarins, *n* (%)	3,540 (3.1)	2,782 (2.8)	758 (6.5)	0.179	1,078 (5.8)	536 (5.7)	542 (5.8)	0.003
Statins, *n* (%)	27,662 (24.6)	24,759 (24.5)	2,903 (24.9)	0.009	4,550 (24.4)	2,262 (24.2)	2,288 (24.5)	0.006
Steroids, *n* (%)	14,214 (12.6)	10,338 (10.2)	3,876 (33.2)	0.581	4,997 (26.8)	2,489 (26.7)	2,508 (26.9)	0.005
NSAIDs, *n* (%)	45,162 (40.1)	38,650 (38.3)	6,512 (55.8)	0.357	9,839 (52.7)	4,932 (52.8)	4,907 (52.6)	0.005
OHAs, *n* (%)	25,343 (22.5)	22,164 (22.0)	3,179 (27.3)	0.124	4,741 (25.4)	2,355 (25.2)	2,386 (25.6)	0.008
Insulins, *n* (%)	24,302 (21.6)	18,256 (18.1)	6,046 (51.8)	0.757	8,515 (45.6)	4,263 (45.7)	4,252 (45.5)	0.002

* *Data are presented as n (%) or medians and interquartile ranges*.

*SMD, standardized mean difference; Hgb, hemoglobin; HbA_1c_, hemoglobin A_1c_; eGFR, estimated glomerular filtration rate; UPCR, spot urine protein-creatinine ratio; HTN, hypertension; CAD, coronary artery disease; CHF, congestive heart failure; COPD, chronic obstructive pulmonary disease; CCI, charlson comorbidity index; CCB, calcium channel blocker; ACEI, angiotensin-converting enzyme inhibitors; ARB, angiotensin receptor blocker; NSAIDs, nonsteroidal anti-inflammatory drugs; OHA, oral hypoglycemic agents*.

### The Risks of All-Cause Mortality, Readmission for AKI, eGFR Decline, and ESRD

In Cox analyses, the sepsis group exhibited greater risks of all-cause mortality [hazard ratio (HR), 1.39; 95% CI, 1.31–1.47; *P* < 0.001], readmission for AKI (HR, 1.67; 95% CI, 1.58–1.76; *P* < 0.001), eGFR decline ≥ 50% or doubling of serum creatinine (HR, 3.34; 95% CI, 2.78–4.01; *P* < 0.001), and ESRD (HR, 1.43; 95% CI, 1.34–1.53; *P* < 0.001; Table 2) compared to the non-sepsis group. The severity of readmission for AKI between sepsis and non-sepsis groups showed as follows: AKIN stage 1: 2,076 (68.5%) sepsis group vs. 1,931 (76.4%) non-sepsis group; AKIN stage 2: 478 (15.8%) sepsis group vs. 320 (12.7%) non-sepsis group; and AKIN stage 3: 477 (15.7%) sepsis group vs. 277 (11.0%) non-sepsis group. In the sepsis group, sepsis (27.9%) was the most common etiology of readmission for AKI followed by cardiogenic causes (24.3%) and nephrotoxic agents (21.3%). In non-sepsis group, cardiogenic causes (31.8%) were the most common etiology followed by nephrotoxic agents (18.0%) and hypovolemia (13.9%).

**Table 2:** Risks of all-cause mortality and adverse renal outcomes between sepsis group and matched non-sepsis group.

**Outcome**	**No. of events**	**Person-years**	**Incidence rate^‡^ (per 100 person-years)**	**Propensity score–matched**		**Competing risk for mortality**	
				**HR (95% CI)**	***P*-value**	**HR (95% CI)**	***P*-value**
**All-cause mortality**							
Non-Sepsis group	2,334	46,180	5.05	Reference		–	–
Sepsis group	2,573	28,607	8.99	1.39 (1.31–1.47)	<0.001	–	–
**Readmission for AKI**							
Non-Sepsis group	2,528	40,277	6.28	Reference		Reference	
Sepsis group	3,031	22,308	13.59	1.67 (1.58–1.76)	<0.001	1.55 (4.36–5.12)	<0.001
**eGFR decline** **≥50% or doubling of serum creatinine**							
Non-Sepsis group	154	45,591	0.34	Reference		Reference	
Sepsis group	467	27,491	1.70	3.34 (2.78–4.01)	<0.001	3.23 (14.74–48.01)	<0.001
**ESRD** ^‡^							
Non-Sepsis group	1,499	40,651	3.69	Reference		Reference	
Sepsis group	1,930	23,997	8.04	1.43 (1.34–1.53)	<0.001	1.39 (3.67–4.42)	<0.001

‡ *End-stage renal disease was defined as an eGFR < 15 ml/min/1.73m^2^, or the initiation of long-term dialysis, or kidney transplantation*.

*No., numbers; HR, hazard ratio; CI, confidence interval; AKI, acute kidney injury; eGFR, estimated glomerular filtration rate; ESRD, end-stage renal disease*.

**Figure 2:**
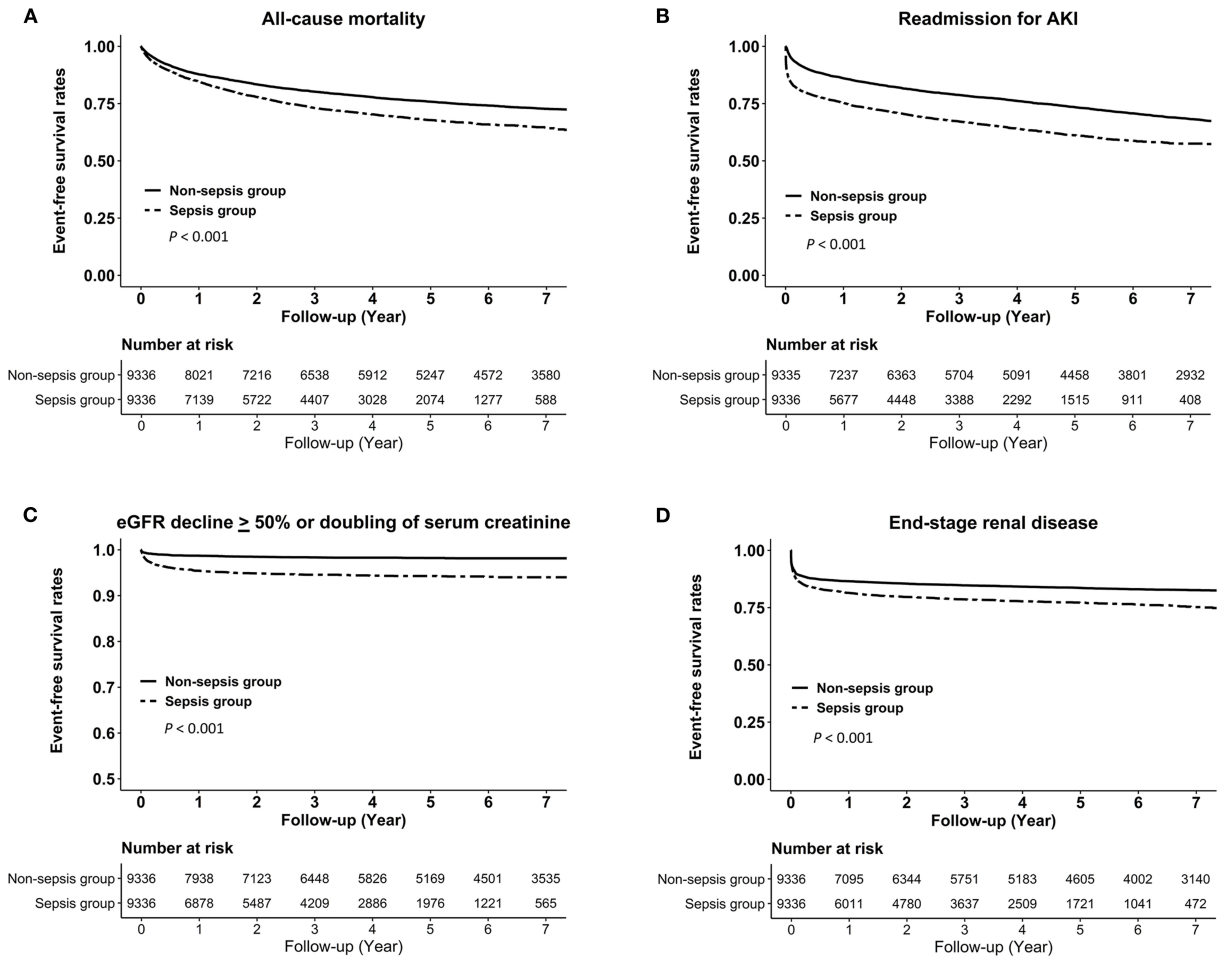
Kaplan–Meier curves for the risks of **(A)** all-cause mortality, **(B)** readmission for AKI, **(C)** eGFR decline > 50% or doubling of serum creatinine, and **(D)** end-stage renal disease in the sepsis group vs. the non-sepsis group. AKI, acute kidney injury; eGFR, estimated glomerular filtration rate.

Kaplan–Meier analysis also showed that the sepsis group was more likely to be at higher risks of all-cause mortality, readmission for AKI, eGFR decline ≥ 50% or doubling of serum creatinine, and ESRD (all log-rank test, *P* < 0.001; Figure 2).

### Competing Risks Analyses With Mortality Considered as a Competing Event

After considering mortality as a competing risk, sepsis group still exhibited higher risks of readmission for AKI (HR, 1.55; 95% CI, 4.36–5.12; *P* < 0.001), and eGFR decline ≥ 50% or doubling of serum creatinine (HR, 3.23; 95% CI, 14.74–48.01; *P* < 0.001), and ESRD (HR, 1.39; 95% CI, 3.67–4.42; *P* < 0.001) compared to the non-sepsis group (Table 2).

### The Subgroup Analyses for the Risks of All-Cause Mortality and Renal Adverse Outcomes

In the subgroup analysis stratified by the eGFR > 60 ml/min/1.73 m^2^ and eGFR < 60 ml/min/1.73 m^2^, the effects of sepsis on all-cause mortality (*P* for interaction = 0.742), readmission for AKI (*P* for interaction = 0.776), eGFR decline ≥ 50% or doubling of serum creatinine (*P* for interaction = 0.894), and ESRD (*P* for interaction = 0.863) were consistent across patient subgroups (Table 3). The results still showed similar after considering mortality as a competing risk.

**Table 3:** Risks of all-cause mortality and adverse renal outcomes between sepsis group and matched nonsepsis group stratified by eGFR > 60 ml/min/1.73 m^2^ and eGFR < 60 ml/min/1.73 m^2^.

**Outcome**	**No. of events**	**Person-years**	**Incidence rate^‡^ (per 100 person-years)**	**Propensity score–matched**		**Competing risk for mortality**	
				**HR (95% CI)**	***P*-value**	**HR (95% CI)**	***P*-value**
**Patients with eGFR** **≥60 ml/min/1.73 m**^**2**^							
**All-cause mortality** ^**§**^							
Non-Sepsis group	905	23,245	3.89	Reference		–	–
Sepsis group	1,197	15,267	7.84	1.58 (1.45–1.73)	<0.001	–	–
**Readmission for AKI** ^**∥**^							
Non-Sepsis group	907	21,584	4.20	Reference		Reference	
Sepsis group	1,209	12,912	9.36	1.85 (1.70–2.03)	<0.001	1.69 (4.73–6.29)	<0.001
**eGFR decline** **≥50% or doubling of serum creatinine**^**¶**^							
Non-Sepsis group	77	22,927	0.34	Reference		Reference	
Sepsis group	243	14,694	1.65	3.33 (2.57–4.30)	<0.001	3.21 (12.02–63.66)	<0.001
**ESRD** ^◇^ ^‡^							
Non-Sepsis group	140	22,838	0.61	Reference		Reference	
Sepsis group	315	14,649	2.15	2.60 (2.12–3.18)	<0.001	2.43 (7.34–19.53)	<0.001
**Patients with eGFR** **<** **60 ml/min/1.73 m**^**2**^							
**All-cause mortality** ^**§**^							
Non-Sepsis group	1,429	22,935	6.23	Reference		–	-
Sepsis group	1,376	13,340	10.31	1.29 (1.19–1.39)	<0.001	–	-
**Readmission for AKI** ^**∥**^							
Non-Sepsis group	1,621	18,692	8.67	Reference		Reference	
Sepsis group	1,822	9,395	19.39	1.64 (1.53-1.75)	<0.001	1.53 (4.19-5.13)	<0.001
**eGFR decline** **≥50% or doubling of serum creatinine**^**¶**^							
Non-Sepsis group	77	22,664	0.34	Reference		Reference	
Sepsis group	224	12,798	1.75	3.34 (2.57-4.33)	<0.001	3.23 (12.13-65.31)	<0.001
**ESRD** ^◇^ ^‡^							
Non-Sepsis group	1,359	17,813	7.63	Reference		Reference	
Sepsis group	1,615	9,348	17.28	1.41 (1.31-1.51)	<0.001	1.37 (3.58-4.36)	<0.001

§ *P for interaction = 0.742*.

∥ *P for interaction = 0.776*.

¶ *P for interaction = 0.894*.

◇ *P for interaction = 0.863*.

‡ *End-stage renal disease was defined as an eGFR < 15 ml/min/1.73 m^2^, or initiation of long-term dialysis, or kidney transplantation*.

*No., numbers; HR, hazard ratio; CI, confidence interval; AKI, acute kidney injury; eGFR, estimated glomerular filtration rate; ESRD, end-stage renal disease*.

### Risk Factors for All-Cause Mortality and Adverse Renal Outcomes

As shown in Table 4, a higher length of hospital stay and SOFA score were associated with higher risks of all-cause mortality, readmission for AKI and ESRD. Across the different etiologies of sepsis, there were similarly increased risks for all-cause mortality, readmission for AKI and ESRD. Based on the severity of sepsis, patients with septic shock had highest risks of all-cause mortality (HR, 2.85; 95% CI, 2.16–3.67; *P* < 0.001), readmission for AKI (HR, 2.81; 95% CI, 2.17–3.57; *P* < 0.001), and ESRD (HR, 8.24; 95% CI, 6.12–10.85; *P* < 0.001) compared to patients with sepsis only. Patients with severe sepsis still had higher risks of all-cause mortality (HR, 1.35; 95% CI, 1.27–1.43; *P* < 0.001), readmission for AKI (HR, 1.48; 95% CI, 1.40–1.56; *P* < 0.001), and ESRD (HR, 7.87; 95% CI, 7.22–8.58; *P* < 0.001) compared to patients with sepsis only. In addition, sepsis patients with AKIN stage 3 exhibited greatest risks of all-cause mortality (HR, 2.61; 95% CI, 1.96–3.40; *P* < 0.001), readmission for AKI (HR, 4.91; 95% CI, 3.88–6.11; *P* < 0.001), and ESRD (HR, 2.94; 95% CI, 2.15–3.90; *P* < 0.001) compared to those with other AKIN stage or those without AKI.

**Table 4:** Risk factors for all-cause mortality and adverse renal outcomes.

**Variables during sepsis admission**	**All-cause mortality**		**Readmission for AKI**		**ESRD^†^**	
	**HR (95% CI)**	***P*-value**	**HR (95% CI)**	***P*-value**	**HR (95% CI)**	***P*-value**
Length of hospital stay (days)	1.01 (1.00–1.01)	<0.001	1.01 (1.00–1.01)	<0.001	1.02 (1.01–1.02)	<0.001
SOFA score	1.17 (1.15–1.19)	<0.001	1.09 (1.07–1.11)	<0.001	1.65 (1.62–1.68)	<0.001
**Infection sources**						
Bacteremia	1.52 (1.24–1.84)	<0.001	1.05 (0.83–1.31)	0.652	1.92 (1.55–2.35)	<0.001
CNS infection	1.51 (1.03–2.12)	0.026	2.04 (1.48–2.74)	<0.001	1.44 (0.92–2.14)	0.087
Endocarditis	1.58 (1.08–2.22)	0.013	1.51 (1.02–2.13)	0.027	2.51 (1.74–3.48)	<0.001
Genitourinary infection	1.31 (1.16–1.47)	<0.001	1.78 (1.60–1.97)	<0.001	1.21 (1.05–1.40)	0.007
Intraabdominal infection	1.57 (1.43–1.71)	<0.001	1.50 (1.37–1.64)	<0.001	1.63 (1.47–1.81)	<0.001
Respiratory infection	1.66 (1.55–1.78)	<0.001	1.71 (1.60–1.84)	<0.001	1.46 (1.34–1.59)	<0.001
**Severity of sepsis**						
Sepsis only	References		References		References	
Severe sepsis	1.35 (1.27–1.43)	<0.001	1.48 (1.40–1.56)	<0.001	7.87 (7.22–8.58)	<0.001
Septic shock	2.85 (2.16–3.67)	<0.001	2.81 (2.17–3.57)	<0.001	8.24 (6.12–10.85)	<0.001
**Severity of AKI**						
No AKI	References		References		References	
AKIN stage 1	1.81 (1.62–2.02)	<0.001	2.25 (2.03–2.48)	<0.001	1.75 (1.53–1.98)	<0.001
AKIN stage 2	1.97 (1.51–2.50)	<0.001	3.06 (2.47–3.74)	<0.001	1.96 (1.47–2.56)	<0.001
AKIN stage 3	2.61 (1.96–3.40)	<0.001	4.91 (3.88–6.11)	<0.001	2.94 (2.15–3.90)	<0.001

† *End-stage renal disease was defined as an eGFR < 15 ml/min/1.73 m^2^, or the initiation of long-term dialysis, or kidney transplantation*.

*AKI, acute kidney injury; ESRD, end-stage renal disease; HR, hazard ratio; CI, confidence interval; SOFA score, sequential organ failure assessment score; CNS, central nervous system; AKIN, acute kidney injury network*.

## Discussion

This large-scale cohort study of 112,628 patients with CKD found that ~10.4% of the patients experienced at least one event of sepsis hospitalization during a long follow-up period. We demonstrated that CKD patients with sepsis had a higher risk of mortality than those without sepsis. In addition, we found that patients with CKD who were discharged from hospitalization for sepsis demonstrated higher risks of readmission for AKI, eGFR decline ≥ 50% or doubling of serum creatinine, and ESRD compared to those without sepsis.

A study including 25,675 participants from a single Canadian health region found that CKD patients with an eGFR of 45–59, 30–44, and <30 ml/min/1.73 m^2^ were at greater risk of bloodstream infection, with hazard ratios (HRs) of 1.24, 1.59, and 3.54 compared to those with a higher eGFR (>60 ml/min/1.73 m^2^) (32). The Atherosclerosis Risk in Communities Study, which included 9,697 participants, also found that those with an eGFR of 15–29 ml/min/1.73 m^2^ had a 3.5-fold higher risk of infection than those with an eGFR > 90 ml/min/1.73 m^2^ (33). Interestingly, another nationwide population study including 62,872 patients with advanced CKD found that those who had an infection before starting dialysis were at increased risk of mortality and major adverse cardiac events compared to those who had no infection (34).

A 1 year follow-up retrospective study including 1,636 patients with sepsis found that ~61% of patients developed AKI during admission. Among these patients, ~19% developed CKD 1 year later, and 81% of patients recovered renal function (35). However, this study was limited only to include patients who had AKI during hospitalization. Whether this result can be generalized to those without AKI is unknown. In addition, the period of only 1 year may also be too short to assess whether AKI resolves or progresses to ESRD. There remains a lack of information regarding the impacts of sepsis on the future risks of renal adverse outcomes, with a particular lack of data in patients with CKD. Our study found that patients with CKD who survived to discharge from sepsis had increased risks of readmission for AKI, worsened renal function decline, and incidence of ESRD compared to patients with CKD without sepsis. Our study found that patients with CKD who suffered from septic shock or severe sepsis during admission were associated with the worst outcomes compared to those with only sepsis. In addition, CKD patients with sepsis who experienced AKI episodes with AKIN stage 3 in their admission had the worst long-term clinical outcomes compared to those with other AKIN stages or those without AKI. In the subgroup analyses, we examined whether the risks varied across patient subgroups stratified by eGFR > 60 or eGFR < 60 ml/min/1.73 m^2^, and results showed no significant effect modification of eGFR.

The possible explanations for the impact of sepsis on worsened renal outcomes are likely to be multifactorial. Sepsis may trigger inflammatory cascades through the release of inflammatory mediators, and the upregulation of reactive oxygen species may induce DNA damage and protein structure alteration and trigger fibrogenic processes, resulting in kidney injury and CKD development (36–38). In addition, sepsis and hemodynamic instability may contribute to acute tubular necrosis and glomerular injury resulting from deposition of circulating immune complexes, which cause macrophage infiltration and oxidative stress damage (39–41). However, further research is still needed to confirm the precise mechanisms of the aforementioned multifaceted mechanisms in such patients.

This study has several important strengths. First, we removed patients with CKD who had fewer than two eGFR measurements, which may provide more precise information on renal function decline. Second, this study was the first to explore the effects of sepsis on long-term renal adverse outcomes in a large number of CKD patients with a long follow-up period, which proved to be important for filling existing knowledge gaps.

Although this study provides information on the relationship between sepsis and renal function decline in patients with CKD, several potential limitations should be noted. First, we excluded patients with CKD who died during hospitalization for sepsis. Therefore, patients with CKD needed to survive to discharge to be included in our analysis. Second, we defined sepsis only by hospitalization events. Therefore, patients with CKD receiving outpatient care for mild sepsis would not be included in our analysis, which may underestimate sepsis rates. However, the clinical presentation of mild sepsis may be non-specific and difficult to differentiate from other diseases, which may lead to a misclassification bias. Finally, this was a retrospective and observational study that may have covariate imbalances among CKD patients with and without sepsis. Therefore, we calculated propensity scores to balance the covariate distributions.

In conclusion, CKD patients with sepsis showed a higher risk of eGFR decline and ESRD than those without sepsis. Therefore, early intervention strategies for patients with CKD who survive hospitalization for sepsis may help to improve long-term renal outcomes and reduce the burden on healthcare systems.

## Data Availability Statement

The raw data supporting the conclusions of this article will be made available by the authors, without undue reservation.

## Ethics Statement

The studies involving human participants were reviewed and approved by Taipei Veterans General Hospital (2017-09-002BC). The Ethics Committee waived the requirement of written informed consent for participation.

## Author Contributions

S-MO, K-HL, M-TT, W-CT, Y-CC, and D-CT: conception, study design, and drafting of the manuscript. S-MO, Y-CC, and D-CT: data acquisition, data analysis/interpretation, and statistical analysis. All authors contributed to the article and approved the submitted version.

## Funding

This study was supported in part by the Ministry of Science and Technology, Taiwan (MOST 106-2314-B-010-039-MY3, MOST 107-2314-B-075-052, MOST 108-2314-B-075-008, MOST 109-2314-B-075−067-MY3, MOST 109-2320-B-075-006, MOST 109-2314-B-075-097-MY3, MOST 110-2312-B-075-002, MOST 110-2634-F-A49-005, and MOST 110-2320-B-075-004-MY3); the Taipei Veterans General Hospital (V107B-027, V108B-023, V108C-103, V108D42-004-MY3-2, V109B-022, V109C-114, V109D50-001-MY3-1, V109D50-001-MY3-2, V109D50-001-MY3-3, V109D50-002-MY3-3, V109E-008-5(110), V110C-152, V110E-003-2, V110E-003-2, V111E-002-3, V111C-171,V111C-151, and V111D60-004-MY3-1); the Taipei Veterans General Hospital, National Yang-Ming University Excellent Physician Scientists Cultivation Program (No.104-V-B-044). Taipei, Taichung, Kaohsiung Veterans General Hospital, Tri-Service General Hospital, Academia Sinica Joint Research Program (VTA110-V1-3-1), and the Foundation for Poison Control (FPC-109-002). The funders did not play any role in the study design, data collection or analysis, decision to publish, or preparation of the manuscript.

## Conflict of Interest

The authors declare that the research was conducted in the absence of any commercial or financial relationships that could be construed as a potential conflict of interest.

## Publisher's Note

All claims expressed in this article are solely those of the authors and do not necessarily represent those of their affiliated organizations, or those of the publisher, the editors and the reviewers. Any product that may be evaluated in this article, or claim that may be made by its manufacturer, is not guaranteed or endorsed by the publisher.
