# Supplementary material for: Sepsis and the Risks of Long-Term Renal Adverse Outcomes in Patients With Chronic Kidney Disease
Source: Front Med (Lausanne). 2022 Feb 24;9:809292. doi: 10.3389/fmed.2022.809292 (PMC8908417; doi:10.3389/fmed.2022.809292)

Sepsis and the risks of long-term renal adverse outcomes in patients with chronic kidney disease

Authors: Shuo-Ming Ou, Kuo-Hua Lee, Ming-Tsun Tsai, Wei-Cheng Tseng, Yuan-Chia Chu, Der-Cherng Tarng

Table of Contents

**Supplemental Tables**	
Supplementary Table 1. Propensity score model results of probability of diagnosis of sepsis among patients with chronic kidney disease	2
**Supplemental Figures**	
Supplementary Figure 1. Covariate balance before and after propensity score matching	3
Supplementary Figure 2. Distributional balance for propensity score before and after matching	4

Supplementary Table 1. Propensity score model results of probability of diagnosis of sepsis among patients with chronic kidney disease

Parameter	Estimate	Odds Ratios	95% CI Lower	95% CI Upper	P value
**Age**	0.019	1.019	1.017	1.021	<0.001
**Male sex**	-0.278	0.757	0.716	0.801	<0.001
**Smokers**	0.390	1.476	1.383	1.576	<0.001
**Alcohol consumption**	0.438	1.550	1.448	1.659	<0.001
**Hgb**	-0.389	0.678	0.669	0.686	<0.001
**Total cholesterol**	-0.004	0.996	0.996	0.997	<0.001
**HbA_{1c}**	0.001	1.001	1.000	1.001	<0.001
**eGFR**	0.005	1.005	1.004	1.006	<0.001
**UPCR**	0.001	1.000	1.000	1.000	0.975
**HTN**	0.766	2.150	2.030	2.277	<0.001
**Diabetes mellitus**	-0.014	0.986	0.932	1.044	0.628
**CAD**	0.227	1.254	1.179	1.335	<0.001
**CHF**	0.678	1.970	1.836	2.114	<0.001
**Peptic ulcer disease**	0.845	2.327	2.184	2.480	<0.001
**COPD**	0.697	2.008	1.864	2.163	<0.001
**Malignancy**	0.760	2.138	2.027	2.256	<0.001
**CCI score**	0.041	1.042	1.031	1.054	<0.001
**CCBs**	0.136	1.146	1.085	1.209	<0.001
**Beta blockers**	0.086	1.089	1.028	1.155	0.004
**Alpha blockers**	0.042	1.043	0.979	1.112	0.192
**ACEIs/ARBs**	-0.214	0.808	0.764	0.853	<0.001
**Antiplatelets**	0.020	1.020	0.964	1.081	0.489
**Warfarins**	0.268	1.307	1.174	1.455	<0.001
**Statins**	-0.101	0.904	0.852	0.959	0.001
**Steroids**	0.911	2.486	2.352	2.627	<0.001
**NSAIDs**	0.445	1.560	1.488	1.636	<0.001
**OHAs**	-0.104	0.902	0.848	0.959	0.001
**Insulins**	1.147	3.149	2.985	3.322	<0.001

Abbreviations: Hgb, hemoglobin; HbA_{1c}, hemoglobin A_{1c}; eGFR, estimated glomerular filtration rate; UPCR, spot urine protein-creatinine ratio; HTN, hypertension; CAD, coronary artery disease; CHF, congestive heart failure; COPD, chronic obstructive pulmonary disease; CCI, Charlson Comorbidity Index; CCB, calcium channel blocker; ACEI, angiotensin converting enzyme inhibitors; ARB, angiotensin receptor blocker; NSAIDs, nonsteroidal anti-inflammatory drugs; OHA, oral hypoglycemic agents.

Supplementary Figure 1. Covariate balance before and after propensity score matching

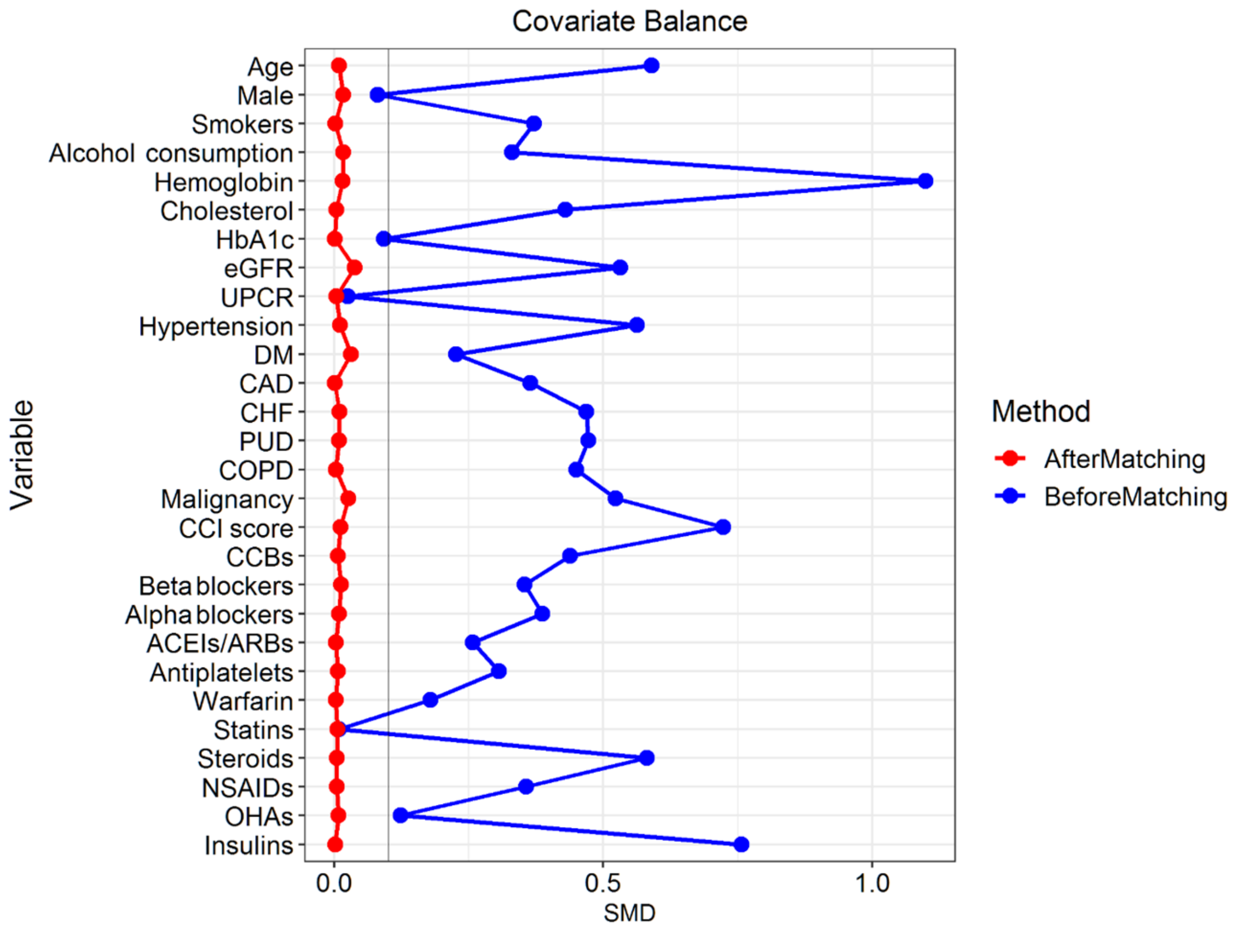

Supplementary Figure 2. Distributional balance for propensity score before and after matching

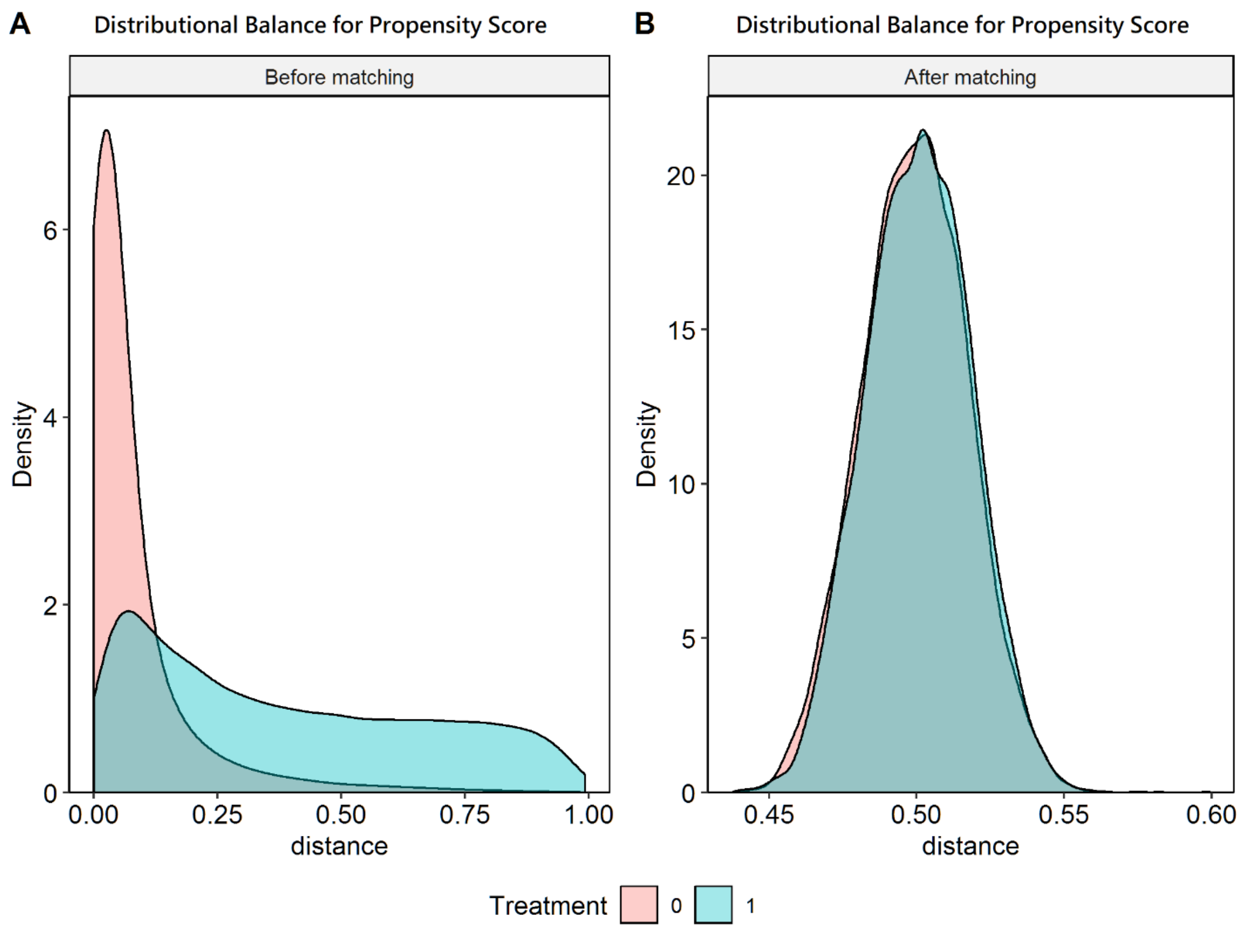

Supplement: Supplementary file 1 [file Data_Sheet_1.pdf]
